# Experiences of managing a gluten-free diet on multiple levels of society: a qualitative study

**DOI:** 10.1186/s40795-020-00390-3

**Published:** 2020-11-23

**Authors:** Lisa Garnweidner-Holme, Karla Sende, Monica Hellmann, Christine Henriksen, Knut E. A. Lundin, Mari C. W. Myhrstad, Vibeke H. Telle-Hansen

**Affiliations:** 1 Department of Nursing and Health Promotion, Faculty of Health Sciences, P.O. 4, St. Olavs Plass, 0130 Oslo Metropolitan University, Oslo, Norway; 2 Det Glutenfrie Verkstedet, Nordseterveien 26A, 1176 Oslo, Norway; 3 Department of Nutrition, Institute of Basic Medical Sciences, University of Oslo, Boks 1072 Blindern, 0316 Oslo, Norway; 4 K. G. Jebsen Coeliac Disease Research Centre, University of Oslo, Boks 1072 Blindern, 0316 Oslo, Norway; 5 Department of Gastroenterology, Oslo University Hospital, Boks 1072 Blindern, 0316 Oslo, Norway

**Keywords:** Coeliac disease, Gluten-free diet, Socio-ecological model, Qualitative, Interpretative phenomenological analysis

## Abstract

**Background:**

Coeliac disease (CD) is an immune-mediated enteropathy against dietary gluten. The treatment for CD is a strict life-long gluten-free (GF) diet, which has a profound effect on a person’s life. In recent years, there has been an increase in the availability of gluten-free products. This study investigates how people with CD experience and manage a GF diet.

**Methods:**

Semi-structured, individual interviews were conducted in different areas of Norway. The analysis was guided by Interpretative Phenomenological Analysis. Participants with CD (*n* = 12) varied in terms of gender, age, family composition and time since diagnosed.

**Results:**

The analysis revealed challenges for a GF diet at the individual, interpersonal, community and policy levels. At the individual level, the participants explained that it took time to gain knowledge about a GF diet, and they expressed uncertainty about the healthiness of a GF diet. At the interpersonal level, the feeling of being different and the fear of gluten contamination were barriers to the enjoyment of social meals. At the community level, the participants asked for a wider selection of tastier GF products to purchase and increased knowledge about CD among those who prepare and sell GF foods. At the policy level, the participants asked for political action to make GF products more affordable.

**Conclusions:**

This study indicates that people with CD should be given information about how to manage a GF diet right after being diagnosed with CD. The food industry should be encouraged to produce healthy and tasty GF products.

**Supplementary Information:**

**Supplementary information** accompanies this paper at 10.1186/s40795-020-00390-3.

## Background

Coeliac disease (CD) is an immune-mediated enteropathy against dietary gluten present in wheat, rye and barley [1]. The prevalence of CD has increased worldwide, making CD one of the most common lifelong food-related disorders [1]. In Norway, the prevalence of CD is estimated to be 1–2%, which means that 50,000–100,000 people have the disease in Norway [2]. The only currently available treatment for CD is a strict adherence to a gluten-free (GF) diet; however, the diet is restrictive and gluten is difficult to avoid [3]. Even small amounts of gluten (i.e. 50 mg) can be harmful to persons with CD [4], an amount that should be compared with the daily intake of approximately 15,000 mg in the Western diet [5]. Untreated CD is associated with gastro-intestinal symptoms and nutritional deficiencies, most of which can be avoided with a strict GF diet [6].

However, following a GF diet has some disadvantages compared to a gluten-containing (GC) diet. A qualitative study among 17 adults diagnosed with CD in Canada concluded that the sole medical recommendation of a GF diet fails to acknowledge the difficulties those with CD can endure in the current GF landscape [7]. Studies show that GF products are more expensive than their GC counterparts [8–10] and that GF products often have a lower nutritional quality than their GC equivalents [11, 12].

People living with CD often experience a lower quality of life compared to the general population [13]. A case-control study in the United Kingdom showed that quality of life in persons with CD is determined by the perceived degree of difficulty adhering to a GF diet [14]. A qualitative study among 43 informants in Sweden identified dilemmas for persons with CD in five arenas: the food situation at work, during purchases, when travelling, in relation to meals at home and when eating meals outside the house [15]. Several studies have focused particularly on how the condition and the GF diet affect social life [7, 15, 16]. In-depth interviews with 10 families in the United States identified social isolation and misunderstandings about CD as the most significant barriers to GF diet adherence [17].

Only a few studies have investigated experiences with a GF diet at multiple levels of society [15]. The social ecological model (SEM) [18] addresses determinants of food choices on the following four societal levels: the individual level (e.g. preferences, knowledge, skills, motivation, attitudes, self-efficacy and self-confidence); the interpersonal level (e.g. family, parenting and personal relationships, social networks and peer pressure/support); the community and organisational level (e.g. food environment, workplaces and educational settings, retail, service, community and recreational facilities) and the international and national public policy level (e.g. policies and subsidies, taxation, advertisement and marketing regulations, food safety, nutrition labelling and food claims, urban planning, food system and supply). The SEM has been widely applied for understanding, exploring and addressing determinants of health at many levels [18]. As the number of persons with CD increases worldwide, the SEM may be useful for making health professionals, policymakers and the food industry aware of the challenges individuals with CD face in a rapidly changing food market.

As in other high-income countries, the availability of GF products is increasing in Norway. In addition, a GF diet has become popular among persons without CD. Thus, this study explores how people with CD experience and manage a GF diet in a changing GF landscape.

## Methods

The interpretative phenomenological analysis (IPA) inspired the research process. As this methodology is suitable for exploring individuals’ perspectives and experiences [19], it was considered appropriate for exploring the experiences of individuals with CD. IPA has previously been used to investigate experiences of CD in a GF-food environment [7].

### Selection of participants and recruitment

Twelve participants were recruited purposively based on the following inclusion criteria: diagnose of CD > 18 years and no other diet-related disorders. Efforts were made to recruit a heterogenic study sample in terms of gender, age, years since diagnosed with CD and residence. The majority of the participants (*n* = 8) were recruited via a Facebook group administered by the Norwegian CD Association. These participants were asked to contact the second author to arrange an interview. The other four participants were recruited by the second author in her network. The participants were recruited continuously through the research process. Recruitment was carried out until we observed replication of response and no new themes emerged from the interviews [20].

### Characteristics of the participants

Table 1 presents the relevant background information of the participants. Participants varied in terms of age (19–58 years), gender and years since diagnosed with CD. Five participants resided in a rural area with a population of 8000–10,000 inhabitants, and seven participants lived in cities with > 500,000 inhabitants.

**Table 1:** Background information about the participants

Participant	Gender	Years since diagnosed with CD	Residence	Household
1	Female	4	Urban	Family
2	Female	9	Urban	Cohabitant
3	Female	18	Urban	Cohabitant
4	Female	22	Rural	Family
5	Male	1	Rural	Single
6	Female	20	Rural	Cohabitant
7	Male	17	Rural	Shared apartment
8	Male	3	Urban	Family
9	Male	22	Urban	Shared apartment
10	Female	5	Rural	Family
11	Female	23	Urban	Cohabitant
12	Female	16	Urban	Cohabitant

### Interviews

Semi-structured interviews were used for data collection. The themes in the interview guide (appendix 1) were developed by the project group and pilot. Minor adjustments were made after the pilot interview. The main themes in the final interview guide were (1) perceptions of the management of a GF diet and experiences of purchasing GF products, (2) experiences of preparing and eating purchased GF products and (3) experiences of self-preparing GF food. The interviews lasted for 30 to 90 min. The interviewer (K.S.) was assisted by LGH at the first interview who has a lot of experience with qualitative interviews. The interviewers did not have any knowledge of the participants prior to the study. The interviewers did not have any personal experiences with CD, but were interested in the topic. The participants could choose to have the interview at their place of work (*n* = 2), at the home of the interviewer (*n* = 3), at their home (*n* = 5) or at a café (*n* = 2). The interviews were conducted between September and November 2019.

### Analysis

The interviews were recorded and transcribed verbatim. None of the participants asked to read the transcripts. Potential themes and subthemes were discussed within the research team. NVivo (11.0) was used to identify and manage new themes. The analysis was guided by IPA [19] and included the following steps: (1) reading and rereading, (2) initial noting, (3) developing emergent themes, (4) searching for connections between emergent themes and clustering them into subthemes and (5) arranging the subthemes into superordinate themes related to the research questions.

## Results

All the participants claimed to strictly eat a GF diet. In general, the participants perceived that they had learned how to manage a GF diet; however, the analysis revealed challenges and barriers for adherence to the GF diet at the individual, interpersonal, community and policy levels. Table 2 presents sub-themes and super-ordinated themes resulting from the analysis.

**Table 2:** Sub-themes and super-ordinated themes

Super-ordinated themes	Individual level	Interpersonal level	Community level	Policy level
Sub-themes	Knowledge about CD Knowledge about a GF diet Health concerns	Strategies to eat a GF diet in a family context Difficult to eat a GF diet in social contexts Perceived negative attitudes towards GF food by people without CD	The ‘GF trend’ Too little availability and variety of GF products in food stores, restaurants and bakeries Insecurity and mistrust in GF products Lack of knowledge about GF food in restaurants, bakeries and canteens	Worries about too expensive GF products Economic burdens to eat GF food Importance of governmental supplementation Challenges related to GF food labelling

### Individual level

The participants mentioned that it took some time to obtain enough knowledge about CD and learn how to manage a GF diet, as exemplified by a female participant who has had CD for almost 20 years: ‘I don’t envy those who get coeliac disease; in the first years, it was really tough to build knowledge all the time, yes’. (Participant 12).

Following a GF diet required the precise planning of when and what they should eat throughout the day. Most participants had difficulties preparing GF food from scratch at home; baking bread and cakes were perceived as particularly challenging. Participants who had been diagnosed with CD for more than 5 years mentioned that they had managed it after some practice. The participants outlined that it had become easier to obtain knowledge about how to prepare GF food in recent years because of information on the Internet and groups for people with CD in social media.

The participants appeared to be concerned about their health and how to achieve a healthy diet. The analysis revealed a discrepancy between whether they perceived a GF diet as healthy or unhealthy. Some participants stated that they ate more healthily because of their diagnosis, as expressed by a male participant who had had CD for 3 years: ‘In this regard, it [being diagnosed with CD] has been a blessing because all fast and unhealthy food is not an option’. (Participant 8).

Others thought that it was more difficult to have a healthy GF diet. They thought that a GF diet contained too little fibre and too much sugar, salt and starch.

### Interpersonal level

All the participants mentioned the importance of eating with other people. Participants who lived on their own generally perceived to manage a GF diet better than participants who lived with people without CD. Participants who lived with family members discussed different ways of following a GF diet when other family members did not have CD. They either prepared GF meals for themselves and GC meals for their family members, or their family members also ate a GF diet. Participants living with friends without CD often perceived themselves as different, as described by a younger female participant living with friends: ‘I feel like a problem child who needs special care to get food’. (Participant 12).

According to the participants, the main barriers to eating with others were that people without CD often did not like GF products because of their taste or texture or that participants were afraid of gluten contamination when others prepared their food. For instance, one participant described fear of gluten contamination as following: ‘Because I’ve often got the question “how GF does it have to be?” And then I get, “It has to be entirely, completely GF”. And then you feel that they don’t believe what you’re saying’. (Participant 4).

### Community level

Participants who had lived with CD for several years felt that the availability and variety of GF products had improved; however, they also mentioned that the availability might seem limited to individuals who were only recently diagnosed with CD: ‘It’s because I have experienced much worse availability before, so now I think it is very good. But for those who are diagnosed today and suddenly have to change … they don’t think it’s good. So it’s about what you are used to’. (Participant 3).

However, the participants still experienced challenges when attempting to obtain GF products in stores, bakeries, restaurants and canteens. Participants residing in both urban and rural areas explained that they often had to go to several stores to get everything they needed. A woman who had lived with CD for many years and who lived in a big city explained the following: ‘I used to buy food beforehand, because I’ve learned that you don’t always find what you want to have’. (Participant 3).

The participants explained that they always had to buy the same products and felt that all the products tasted the same. They asked for more ready-made meals to be made available: ‘The dream product is bread that won’t get dry after it has been defrosted and that stays fresh when you prepare a sandwich to bring along …. That’s the dream, and it is healthy, so you avoid sugar. That’s my dream.’ (Participant 4).

Separate sections for GF products in the stores gave many of the participants a feeling of being different.

In several interviews, the participants described positive experiences of eating GF food in restaurants, and they were generally satisfied with the GF labelling in restaurants. However, they felt that many employees showed scepticism and mistrust about GF food.

The participants acknowledged the increasing availability and variety of GF products in bakeries. However, mistrust and fear for gluten contamination occurred in this context: ‘It’s more that I think it is scary because they also bake GC products, so I am nervous and I hope that they don’t use the same cutting board and knife and those things. But, if there are bakers who work there, they might have more knowledge than, for example, a small restaurant, so I feel safe, but at the same time a bit sceptical’. (Participant 7).

Participants handled this insecurity and fear of gluten contamination by bringing their own food, as one student described: ‘I would rather bring some food from home than purchase food in the school canteen because then I know that it is safe to eat, and there is a lot more food that I can prepare at home than what I can buy in the canteen’. (Participant 7).

Participants often talked about the ‘GF trend’, which refers to the increase in people without CD eating GF food. They considered that the GF trend increased the availability of more tasty GF products. However, one female participant expressed her concern that this trend would lead to fewer GF products in the stores: ‘GF products have become a very “hype” among people who do not necessarily have CD or a gluten allergy because they want to be healthy. So, I think that it can be a problem that so many people want to have it because there has to be enough products in the stores so that they are not sold out when you go there’. (Participant 2).

Several participants considered the GF trend as a threat to the seriousness of the disease. They were afraid that people would be less cautious about gluten-contamination as GF food became more common among people without CD.

### Policy level

The participants all thought that GF food was too expensive, as a woman who has lived with CD for several years explained: ‘Expensive, it is very expensive. And I think that people who don’t have it [CD] realise it when they are going to buy it, so they think “Oh my God”, but the price is pretty bad’. (Participant 4).

Some participants tried to avoid buying GF products to save money and a few participants accepted the higher price because they thought that it was more difficult to produce GF products. Participants asked for political actions to make a GF diet more affordable: ‘It should not be economically up to the people with CD to stay healthy’. (Participant 11).

The participants had strong concerns about the recent decrease in government subsidies for people with CD. The majority of the participants stated that the current subsidies did not cover the extra costs for a GF diet, leaving them with financial problems. Others thought that the subsidies covered a GF diet based on products naturally free of gluten that they prepared at home, as explained by a young woman: ‘We have received support from the state for many years. And I think that I eat a lot that is naturally GF, so I don’t think that it is that bad when you calculate how much you spend on a GF diet per month’. (Participant 2).

Another theme at the policy level was participants’ experiences with GF labelling regulations. The participants acknowledged easy labelling systems of GF food. However, the participants expressed uncertainty about the labelling ‘may contain traces of’ because they would prefer to know the exact amount of gluten that the product contained.

## Discussion

The analysis revealed challenges to implementing a GF diet at the individual, interpersonal, community and policy levels.

At the individual level, and in line with a previous study [7], the participants claimed that it took some time to obtain enough knowledge about CD and to learn how to prepare and find GF food after being diagnosed with CD. In a Canadian national survey on the impact of a GF diet, 44% of 2681 people with CD had difficulties following the diet [21]. Comparable with our findings, individuals following a GF diet for over 5 years experienced fewer difficulties in a national US survey [22]. Knowledge of how to follow a GF diet and developing coping skills can help to reduce the difficulties of adapting to a GF diet [14]. Receiving information about a GF diet promptly after a diagnosis of CD is important for initiating good dietary adherence, symptom recovery and improving quality of life [16]. The participants in our study who had lived with CD for several years acknowledged the increased and more readily available information about CD in recent years. We have not explored their most important sources for information in detail. Canadians with CD perceived the usefulness of the information they obtained about CD ranged from 90.4% (Coeliac Support Association) to 52.1% (dietitian) and to 25.3% (family doctor) [22].

The participants in our study were concerned about their health, yet they were sometimes uncertain about the healthiness of a GF diet. Some participants perceived that they started to eat more healthily following their diagnosis of CD since they could no longer eat fast food and unhealthy food. Others were afraid that the GF products are unhealthy. Studies comparing the nutritional quality of GF products with their GC counterparts show that GF products contain more salt, sugar and saturated fats and less fibre and protein compared to their GF counterparts [11, 12, 23]. A similar study is currently ongoing in Norway, as there are currently no published data on the nutritional quality of Norwegian GF products. However, given the increased global food marked, these findings are likely transferable to the Norwegian food market.

There is general agreement that CD has an impact not only at the individual level but also at the familial and social systems levels [17]. At the interpersonal level in our study, the feeling of being different and fear of gluten contamination were barriers to eating common meals. In a qualitative study among 43 persons with CD in Sweden, the participants experienced shame about their diet in social situations. They even wanted to isolate themselves when they could not eat the same food as other people [15]. The participants in our study have developed strategies to eat GF food with people without CD. However, they typically found it more difficult to eat with non-family members. In line with other studies, bringing own food to social gatherings was a common strategy to adhere to a GF diet [17].

At the community level, participants residing in both rural and urban areas wanted a wider selection of tastier GF products to purchase. Several international studies have found a low level of satisfaction with the availability and quality of GF products [15, 22, 24, 25]. Dissatisfaction with the availability and quality of GF products is associated with lower adherence to a GF diet [26]. However, eating GF food has also become popular among those not having CD [27]. Participants considered the GF trend as both an opportunity for the increased availability of more tasty GF products and a threat to the seriousness of the disease. King et al. (2019) explored experiences in the growth of the GF industry among 17 persons with CD in Calgary, Canada. The participants in King et al.’s (2019) study experienced the growth of the GF industry as a ‘double-edged sword’. As in our study, the participants acknowledged the increase in more palatable GF options, and they were increasingly faced with misunderstandings about the severity of CD as a result of many non-CD individuals subscribing to the GF diet [7]. In both studies, the participants were unsure whether those preparing their food had sufficient knowledge of a GF diet. According to a 10-year follow-up study conducted in the UK, the awareness of gluten-related disorders among chefs and the public has increased [28]. However, effective communication strategies are necessary to increase knowledge further about CD among those preparing and selling food to prevent gluten-contamination.

At the policy level, the participants asked for political actions to make a GF diet more affordable, although some participants explained that they saved money by preparing food with naturally GF products. Even though individuals with CD receive governmental subsidies in Norway, the majority of the participants was concerned about the high price of GF food in food stores, bakeries and restaurants. The price of GF products was one of the most important barriers to a GF diet in studies from other countries [7, 25]. In contrast to these studies [7, 25], some of our participants accepted the higher price because of the higher costs involved in producing GF products for the food industry.

The participants in our study discussed the labelling system of GF products. Some had difficulties identifying the right products without the GF labelling. Similarly, other studies have proposed better labelling of GC ingredients in food products to improve quality of life [22, 24]. Zarkadas et al. (2013) found that about 30% of participants in a national Canadian survey experienced difficulties understanding the labelling of GF products 5 years after a CD diagnosis [22]. Participants’ strategies to overcome these difficulties included reading every food ingredient list, labelling all GF flours and having snacks on hand at work or school [22].

### Limitations

The study was conducted among a small study sample, which is typical of qualitative studies [19]. Thus, the findings cannot be generalised. However, given the increased globalisation of the food systems, individuals with CD might have similar experiences in other high-income countries. The participants claimed to adhere to a GF diet; however, no dietary assessment was conducted to prove their claims.

## Conclusions

The findings from the present study indicate that people with CD should be provided with information about how to prepare healthy GF foods right after being diagnosed with CD and the food industry should be encouraged to produce healthy and tasty GF products. The participants also asked for political action to make GF products more affordable.

## Supplementary Information

**Additional file 1.**

## Data Availability

The datasets used and analysed during the current study are available from the corresponding author on reasonable request.

## Abbreviations

CD: Coeliac Disease
GF: Gluten-Free
IPA: Interpretative Phenomenological Analysis
SEM: Social Ecological Model
