# Supplementary material for: Experiences of managing a gluten-free diet on multiple levels of society: a qualitative study
Source: BMC Nutr. 2020 Nov 23;6:65. doi: 10.1186/s40795-020-00390-3 (PMC7682064; doi:10.1186/s40795-020-00390-3)
Supplement: Supplementary file 1 — Additional file 1. [file 40795_2020_390_MOESM1_ESM.docx]

**Interview guide**

Introduction

- 1. Welcoming and introduction
  2. Information about the aim of the study
  3. Obtaining informed consent and information about participants’ privacy

Warm-up and qualification of the participants

2.1 Can you present yourself? How old are you, where and with whom do you live? 2.2 When have you been diagnosed with coeliac disease?

Probing questions

3.1 Experiences of purchasing gluten-free products

a) Can you tell about your general experiences with purchasing gluten-free products?

b) Where do you prefer to buy gluten-free products, and can you explain why?

c) How do you experience the variety in the selection of gluten-free products?

3.2 Experiences to prepare and eat purchased gluten-free products

a) Can you tell me about your general experiences with preparing and eating gluten-free products that you have bought?

b) What do you think about the taste of these products?

3.3 Experiences of self-preparing gluten-free food

a) Do you use to prepare gluten-free food from scratch? If yes, can you describe your experiences with it?

b) Can you describe your perceived advantages and disadvantages of preparing gluten-free food from scratch at home?

Conclusion

4.1 Clarifying questions

4.2 Thanking participants for their involvement and concluding discussion
